# Conception and development of a neurological registry of patients with persistent health impairments following work-related COVID-19 disease in Germany

**DOI:** 10.3205/dgkh000517

**Published:** 2024-11-26

**Authors:** Peter Schwenkreis, Agnessa Kozak, Andreas Gonschorek, Ingo Schmehl, Susann Seddigh, Andrea Fürst, Kai Wohlfahrt, Corinna Rademacher, Lynn Engel, Jacob Wefers, Kerrin Kobes, Olaf Kleinmüller, Jana Wischnat, Albert Nienhaus, Martin Tegenthoff

**Affiliations:** 1 BG University Hospital Bergmannsheil, Department of Neurology, Ruhr-University Bochum, Germany; 2 German Social Accident Insurance Institution for the Health and Welfare Services (BGW), Hamburg, Germany; 3 BG hospital Hamburg, Department of Neurology, Hamburg, Germany; 4 BG hospital Unfallkrankenhaus Berlin, Department of Neurology, Berlin, Germany; 5 BG hospital Duisburg, Department of Neurology, Duisburg, Germany; 6 BG hospital Murnau, Department of Neurology, Murnau, Germany; 7 BG hospital Bergmannstrost Halle, Department of Neurology, Halle, Germany; 8 Center for Epidemiology and Health Services Research for Healthcare Professionals (CVcare), University Medical Center Hamburg-Eppendorf (UKE), Hamburg, Germany

**Keywords:** acute post-COVID-19 syndrome, occupational diseases, health personnel, multicenter post-COVID registry

## Abstract

**Background:**

Healthcare and social workers had an increased occupational risk of contracting SARS-CoV-2 during the pandemic. Some developed long-lasting symptoms known as post-COVID syndrome (PCS). To assess the consequences of COVID-19 for individuals insured by the German Social Accident Insurance, the BG hospitals (Berufsgenossenschaftliche Kliniken: clinics for occupational accident insurance) established an interdisciplinary diagnostic programme. Data collected during routine clinical practice are transmitted to a multicenter post-COVID registry to enhance knowledge of the long-term consequences related to COVID-19 and to optimize diagnostics, treatment, and rehabilitation. The design of the post-COVID registry, along with a description of the study population, is detailed in this paper.

**Methods:**

The registry includes patients with an occupational disease or accident. Depending on the severity and complexity of the symptoms, patients received an outpatient post-COVID examination or an inpatient post-COVID check (PCC). The collected data comprise demographics, occupational and social history, disease progression, pre-existing conditions, utilization of health services, persistent symptoms, and psychosocial and neuropsychological assessments. Further investigations are carried out in response to symptoms and needs, using clinical assessment, instrumental and imaging techniques, as well as questionnaires. In addition, serum and cerebrospinal fluid samples are preserved for biomarker analysis.

**Results:**

By September 2024, 1,957 patients from six BG hospitals were included. An interim analysis of 1,150 cases shows that patients are predominantly female (77%) and the average age is 51 years (standard deviation [SD] 10.5). Around 43% worked in nursing at the time of infection. In 63% of cases, an inpatient post-COVID check was carried out. About 20% were hospitalized during acute COVID-19 infection, with an average stay of 14.6 days (SD 18.4). More than half were still unable to work at the time of examination, with no significant differences between hospitalized and non-hospitalized patients. Common pre-existing conditions included heart disease (48%), allergies (45%), and lung disease (33%). PCS symptoms mainly consisted of reduced physical capacity (95%), concentration difficulties (79%), and shortness of breath (69%). 81% had previously received outpatient and/or inpatient rehabilitation.

**Conclusion:**

The outpatient and the inpatient PCC are essential in managing the recovery process for patients with PCS. Data analysis will provide insights into the need for medical care and rehabilitation. In addition, longitudinal analyses will be used to track the progress of the post-COVID registry over time and monitor the effectiveness of the recommended measures.

## Zusammenfassung

**Hintergrund:**

Beschäftigte im Gesundheits- und Sozialwesen hatten während der Pandemie ein erhöhtes berufliches Risiko, sich mit SARS-CoV-2 zu infizieren. Einige entwickelten langanhaltende Symptome, bekannt als Post-COVID-Syndrom (PCS). Zur Abschätzung der Folgen für Versicherte der Deutschen Gesetzlichen Unfallversicherung implementierten die BG-Kliniken ein interdisziplinäres Diagnoseprogramm. Die im Rahmen der klinischen Routine erhobenen Daten fließen in ein multizentrisches Post-COVID-Register ein. Ziel ist es, Langzeitfolgen besser zu verstehen, Diagnostik, Therapie und Rehabilitation zu optimieren und daraus Handlungsempfehlungen für die Rehabilitationsverfahren sowie die Forschung abzuleiten. Der Aufbau des Registers und die Beschreibung der Studienpopulation werden in diesem Beitrag beschrieben.

**Methoden:**

Das Register umfasst Patienten mit arbeitsbedingten Berufskrankheiten oder Unfällen. Je nach Symptomschwere erhalten die Patienten eine ambulante Untersuchung oder einen stationären Post-COVID-Check (PCC). Erfasst werden u.a. demographische Daten, Arbeits- und Sozialanamnese, Krankheitsverlauf, Vorerkrankungen, Inanspruchnahme von Gesundheitsleistungen, persistierende Beschwerden, psychosoziale und neuropsychologische Untersuchungen. Weitere Untersuchungen erfolgen symptomorientiert mittels klinischer, apparativer und bildgebender Verfahren sowie Fragebögen. Zusätzlich werden Serum- und Liquorproben für spätere Biomarker-Analysen asserviert.

**Ergebnisse:**

Bis September 2024 wurden 1.957 Patientinnen und Patienten aus sechs BG-Kliniken einbezogen. Eine Zwischenanalyse von 1.150 Fällen zeigt, dass 77% weiblich sind und das Durchschnittsalter 51 Jahre (SD 10,5) beträgt. 43% waren zum Infektionszeitpunkt in der Krankenpflege tätig. In 63% der Fälle wurde ein stationärer PCC durchgeführt. Etwa 20% wurden akut stationär behandelt mit einer Verweildauer von durchschnittlich 14,6 d (SD 18,4). Mehr als die Hälfte war bei der Untersuchung noch arbeitsunfähig, wobei es keine signifikanten Unterschiede zwischen hospitalisierten und nicht-hospitalisierten Patienten gab. Zu den häufigsten Vorerkrankungen gehörten Herzerkrankungen (48%), Allergien (45%) und Lungenerkrankungen (33%). PCS-Symptomen waren vor allem verminderte Leistungsfähigkeit (95%), Konzentrationsschwierigkeiten (79%) und Kurzatmigkeit (69%). 81% hatten zuvor eine ambulante und/oder stationäre Rehabilitation erhalten.

**Schlussfolgerung:**

Die ambulante Sprechstunde und der PCC sind zentrale Maßnahmen zur Heilverfahrenssteuerung bei PCS-Patienten. Weitere Datenanalysen sollen den medizinischen Versorgungs- und Rehabilitationsbedarf klären. Zur Überprüfung der zeitlichen Entwicklung und der Wirksamkeit der empfohlenen Maßnahmen werden Längsschnittanalysen durchgeführt.

## Background

### Post-COVID syndrome (PCS)

The condition known as post-COVID syndrome (PCS) can develop as a result of infection with SARS-CoV-2 [1], [2]. The World Health Organization (WHO) defines PCS as symptoms that persist, fluctuate or relapse three months after initial recovery from an acute COVID-19 episode and cannot be explained by other diagnoses [3]. This leads to heterogeneous clinical symptoms and organ manifestations. The pathophysiological mechanisms responsible for the persistence or recurrence of symptoms are still largely unknown. A review from 2022 presented numerous potential mechanisms for different clinical symptoms [4]. The key hypotheses mentioned are endothelial dysfunction, viral persistence, autoimmunity triggered by the infection, persistent inflammation and psychosocial factors [5], although the heterogeneity of the symptoms also suggests that the pathophysiological mechanisms are heterogeneous. Many symptoms are documented in the literature [6], [7], [8], [9]. According to a meta-analysis of 76 studies, the most common symptoms after four to six months were exhaustion, shortness of breath, post-exertional malaise (PEM), sleep disorders and depression. PEM, muscle weakness, depression, anxiety, and exhaustion were also common after six months [9]. There is also evidence of symptom clusters following symptomatic SARS-CoV-2 infection. In a meta-analysis of 1.2 million people from 22 countries with persistent PCS, three clusters were identified according to the predominant symptom: persistent respiratory complaints, persistent fatigue with physical pain or mood swings, and cognitive impairment [10]. These symptoms are associated with both severe courses of the disease which require hospitalization, and mild to moderate courses [11], [12]. Various risk factors such as age, sex and weight also exacerbate the course of the disease [1]. Moreover, several pre-existing conditions are associated with an increased risk of developing PCS. People who reported suffering from asthma, chronic constipation, gastrointestinal reflux, rheumatoid arthritis, allergies, depression or anxiety were at significantly increased risk [13]. 

Given the diversity of diagnostic criteria and clinical phenotypes, there is still uncertainty regarding the pathogenesis, prevalence and treatment of PCS [14]. Complex manifestations such as those seen in PCS patients should be investigated in the most differentiated, objective and quantifiable way possible, particularly regarding possible confounders.

### Workers in health and welfare services

Healthcare and social workers were at particular risk of contracting SARS-CoV-2 during the pandemic [15], [16], [17]. According to the German health insurance funds, workers in the healthcare sector were 2.4 times more likely to be incapacitated for work or be hospitalized due to COVID-19 than workers in any other sector [18]. After the acute illness, some healthcare and social workers experienced persistent symptoms that lasted longer than three months [19].

### COVID-19 as an occupational disease or accident 

A work-related illness resulting from infection with the SARS-CoV-2 virus can be recognized as an occupational disease or accident if the legal requirements are met. COVID-19 is considered an occupational disease (OD) according to No. 3101 of Annex 1 of the German Occupational Disease Ordinance (BKV). The prerequisite is that the insured person works either in health services or in welfare services, in a laboratory, or in professions with a similarly increased risk of infection, and that there is a SARS-CoV-2 infection with corresponding symptoms. COVID-19 can also be recognized as a work-related accident if the person affected does not meet the requirements for an infection to be recognized as an OD [20], [21], [22]. By 30 June 2024, the German Social Accident Insurance (DGUV) providers had recognized 359,763 occupational diseases and 27,069 work-related accidents due to COVID-19 [23].

### Post-COVID diagnostic program

**Figure 1:**
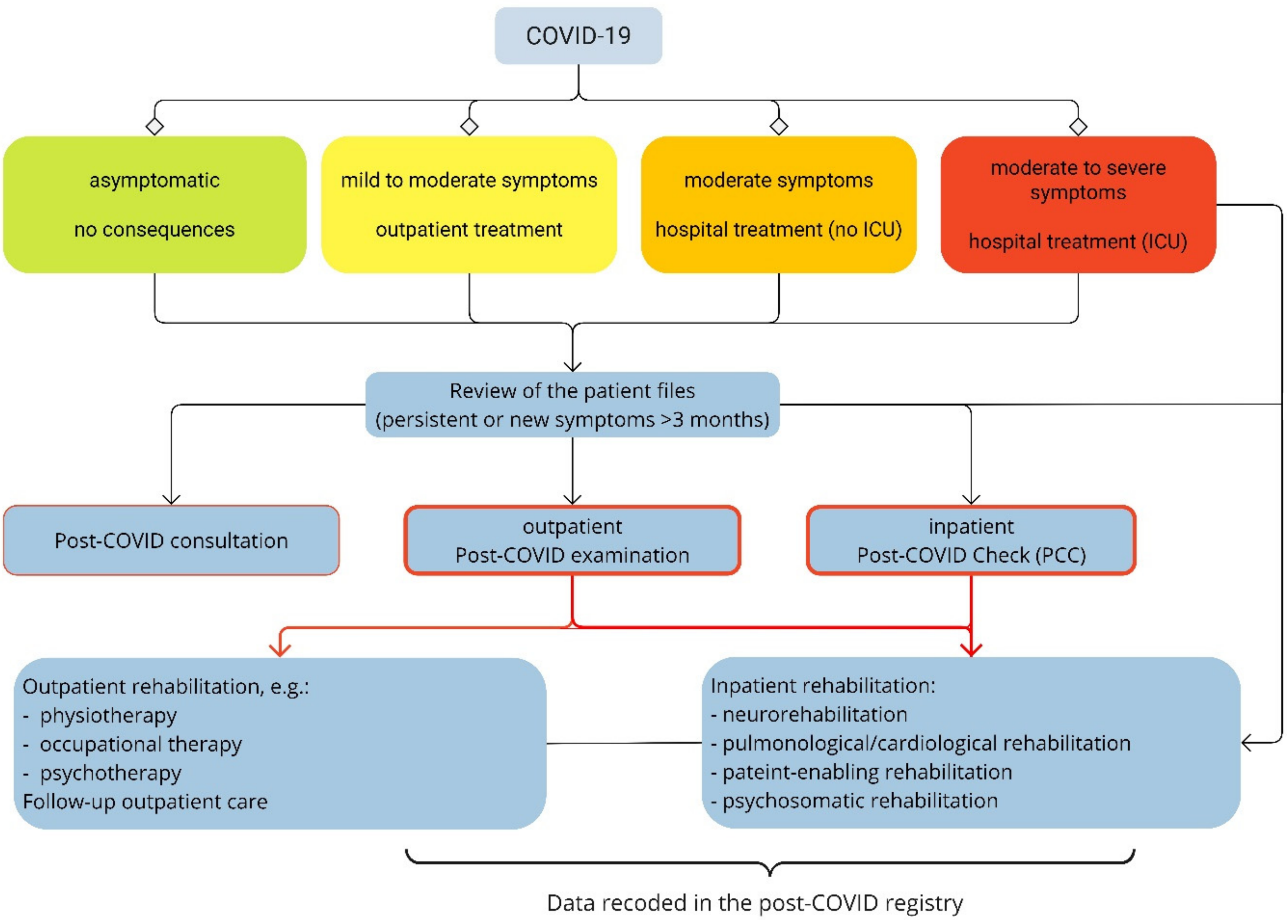
Post-COVID diagnostic program of the BG hospitals

The BG hospitals (clinics for occupational accident insurance) of the DGUV established an interdisciplinary outpatient and inpatient diagnostic program under the leadership of the respective neurological clinic for patients with persistent health issues after work-related COVID-19, in order to better assess the consequences of COVID-19 for persons insured under DGUV (Figure 1 (Fig. 1)). 

The examinations are performed on a symptom- and needs-oriented basis. Patients are categorized as either outpatient or inpatient cases based on the severity of their symptoms. Those with mild symptoms and a positive prognosis for their capacity to work are examined as outpatients, while those with a risk of long-term incapacity for work and complex symptoms as well as with multiple pre-existing and concomitant illnesses are examined as inpatients. The key element of this program is the “post-COVID check” (PCC), which takes place during an inpatient stay. During PCC, patients undergo extensive neurological, neuro-psychological, psychological, physiotherapeutical, ergotherapeutical, pneumological and cardiological examinations. Other disciplines, e.g. otorhinolaryngology, are also consulted when necessary. After this initial inpatient or outpatient diagnostic process in the BG hospitals, the appropriate symptom-oriented treatment or rehabilitation measures are initiated according to the indication. This is followed by inpatient or outpatient follow-up examinations in the hospitals. These measures aim to improve social participation and more importantly, to achieve permanent employment.

In 2021, the BG hospitals’ Neurotrauma Working Group (WG Neurotrauma), in collaboration with the German Social Accident Insurance Institution for the Health and Welfare Services (BGW), initiated the multi-center post-COVID registry, which incorporates data collected during routine clinical care from the PCC and during outpatient examinations. 

### Objectives of the registry 

The aim is to gain further insights into the long-term consequences of COVID-19 and to optimize diagnostics, therapy and rehabilitation of insured patients with PCS. Given the complexity of PCS, this study has several objectives:

- To describe the frequency of subjectively perceived complaints and organ manifestations depending on the time of onset of the disease or vaccination status
- To determine the main characteristic symptoms and their severity, with particular emphasis on the ability to work
- To identify factors associated with persistent symptoms, such as chronic fatigue
- To assess the need for rehabilitation measures for patients with PCS
- To examine neurocognitive functions after mild to severe courses of acute COVID-19 disease
- To determine the extent to which chronic and mental pre-existing conditions aggravate the development of PCS
- To gain insights for case management and the assessment of insured persons with PCS
- To observe the symptoms longitudinally and the effectiveness of outpatient and/or inpatient treatment measures for patients with PCS.

## Method

### Participating facilities 

Six BG hospitals (Berufsgenossenschaftliche Kliniken, BG: clinics for occupational accident insurance – BG University Hospital Bergmannsheil Bochum, BG Hospital Berlin, BG Hospital Duisburg, BG Hospital Bergmannstrost Halle, BG Hospital Hamburg, BG Hospital Murnau) are participating in this multicenter, prospective registry study in a clinical setting. The project is led by the Department of Neurology at the BG University Hospital Bergmannsheil Bochum. The following institutions are also involved in data analysis, data quality management and the establishment of a biobank: the Competence Center for Epidemiology and Health Services Research for Healthcare Professionals (CVcare) at the University Medical Centre Hamburg-Eppendorf (UKE), German Social Accident Insurance Institution for the Health and Welfare Services (BGW), and the Institute for Prevention and Occupational Medicine (IPA) in Bochum. The aim is to record clinical data of the PCC, the initial outpatient examinations, and the inpatient or outpatient follow-up examinations of the BG hospitals in a joint register. The collected data are used for scientific evaluations. 

### Study population 

Patients who are admitted to one of the six participating BG hospitals for further examination as outpatients or inpatients and have symptoms (PCS) that persist for more than 12 weeks after the onset of acute COVID-19 or that have newly appeared are included in the registry. The prerequisite is an infection with SARS-CoV-2 acquired in the workplace (occupational disease 3,101 or work-related accident) and confirmed by a positive PCR, antibody or antigen test. The registry includes patients aged 18 and older from various professional backgrounds who have consented to the continued processing of the data collected during their clinical examinations. There are no specific exclusion criteria. Since this is an ongoing registry, there is no limit on the number of cases. Based on the documented cases from 2021 at the six participating BG hospitals, it was anticipated that 80 to 100 patients would initially be enrolled in the registry each month.

### Recruitment and follow-up 

#### Prospective recruitment 

The DGUV providers decide on the registration of insured persons for post-COVID diagnostics at the BG hospital as part of rehabilitation management after COVID-19. The decision as to whether patients should undergo an outpatient appointment in the post-COVID clinic or an inpatient stay for the PCC is made by the physicians at the respective hospital after reviewing the patient’s file and in consultation with the statutory accident insurance provider (UVT). Insured persons who visit the participating centers for inpatient or outpatient post-COVID diagnostics are informed about the registry study by the respective study manager, given detailed explanations and asked for their consent to participate. In addition, they are asked to give their consent to their data being processed in pseudonymized form and forwarded to the cooperating partners. Prospective inclusion into the registry began on 17 August 2021. The duration of the registry has been extended to 31 December 2025. A further extension is planned due to the ongoing emergence of new cases of COVID-19. 

### Retrospective recruitment 

Patients who were examined at the participating hospital prior to the establishment of the registry and who had not yet consented to the prospective use of their data were contacted by e-mail or post and asked to give their consent to the scientific use of their clinical data. If consent is given by returning a signed consent form, data already collected will be treated as prospectively enrolled data.

### Follow-up examinations 

Follow-up examinations are carried out on a needs-oriented basis and are not scheduled systematically. In some cases, follow-up examinations are carried out after recommended inpatient rehabilitation measures.

### Clinical examinations 

The examination program is structured in stages. Each examination starts with a detailed anamnesis of demographic data, work and social history. A wide range of questions are asked about the SARS-CoV-2 infection and the course of the disease (Table 1 (Tab. 1)). 

Patients are then examined using a range of clinical, instrumental and imaging techniques as listed in Table 2 (Tab. 2).

### Biobank 

A biological materials repository (serum samples and occasionally cerebrospinal fluid [CSF] samples) has also been set up in a sub-project. This contains biological materials for further analyses. The preservation of biological samples makes it possible to carry out correlation analyses between biomarkers and clinical symptoms at a later stage. For this purpose, serum samples are stored in a biobank at the IPA Bochum, if patients have given their consent. If there is an indication for the collection of CSF as part of clinical routine treatment, the residual CSF not required for clinical analyses is also stored at the biobank. The necessary technical and operational infrastructure for the biobank is in place at the IPA.

### Statistics 

The data is pooled for analysis and initially analysed descriptively. Descriptive statistics include location and dispersion parameters for continuous variables, as well as frequencies and percentages for categorical variables. The group differences are determined using parametric or non-parametric tests, depending on the research question, distribution, and data level. Multivariate regression analyses are performed to analyse the relationships between clinical characteristics and outcome variables. Mixed-effects models are used to control for possible differences between the centers, with the centers modelled as random effects. This allows both intra- and inter-center variations to be considered, which leads to robust estimates of the parameters being tested. Appropriate sensitivity and subgroup analyses are carried out to assess the validity and robustness of the results (including the effects of missing data, analyses based on center, visit [outpatient or PCC], age, sex, and severity of the acute course).

### Technologies used 

The browser-based software Research Electronic Data Capture (REDCap) was used to collect and manage the study data. REDCap is hosted by the University Medical Centre Hamburg-Eppendorf. This software was developed by Vanderbilt University also for clinical translational research databases [24]. IBM SPSS Statistics for Windows version 28.0 was used to process and analyse the data. 

### Ethical considerations 

The study protocol complies with the ethical principles of the Declaration of Helsinki [25]. The ethics committee of the Medical Faculty of Ruhr University Bochum provided advice in accordance with national statutory requirements and the ICH GCP guidelines [26]. The application dated 15 July 2021 was confirmed by the ethics committee on 17 August 2021 (registry no. 21-7317). Following the vote of the main ethics committee, the local ethics committees of the other five centers were consulted in an advisory capacity. To include data from patients seen prior to the start of prospective enrolment in the PCC registry, we also submitted an amendment to the ethics application for retrospective enrolment to the relevant ethics committee, which was approved on 16 March 2022. Local ethics committees have also been consulted.

### Data protection considerations 

The data collected in this project are processed under the joint data protection responsibility of the parties involved. Each hospital must ensure that data are handled securely within their respective area of responsibility in accordance with a contractual agreement concluded pursuant to Art. 26 of the General Data Protection Regulation (GDPR). In particular, the protection of the personal data of participants from arbitrary or involuntary identification is guaranteed. The data protection supervisor of the BG hospitals and the University Medical Center Hamburg-Eppendorf were consulted in an advisory position.

### Pseudonymisation 

All patients can be identified only by an individual eight-digit pseudonymization code. For this purpose, each center keeps a list of numbers randomly generated by Matrix Laboratory (MATLAB), which assigns consecutively. Each study center maintains a patient identification log, which is stored on a secure server with restricted access. It is therefore only possible for the local study center to assign patient IDs to the study participants. 

### Handling registry data and biological samples 

The data are entered into REDCap at the study centers in pseudonymized form as an electronic Clinical Report Form (eCRF) and stored on a server at the UKE in Hamburg. Only personnel authorized by the study management have access to the data. The biological samples are preserved for a maximum of 10 years after the time of sampling according to standardized quality and safety measures. 

## Results

By September 2024, 94% of patients screened in the post-COVID program from the six participating BG hospitals had consented to further use of their clinical data and were therefore included in the registry (n=1,957). The majority of patients (72%) were recruited from clinics in Hamburg, Bochum, and Berlin (Table 3 (Tab. 3)). As recruitment is still ongoing and not all cases (backlog) have been entered into REDCap, this descriptive analysis is based on a sub-sample of 1,150 cases. Also, a total of 460 serum and 18 CSF samples were obtained and stored in the biobank. In most cases (63%), an inpatient PCC was performed. The average time between the first SARS-CoV-2 infection and presentation at an inpatient PCC or outpatient examination was 17.4 months (SD 7.8). 

Occupational diseases accounted for 64% and accidents at work for 25% of the sample population (Table 4 (Tab. 4)). Most patients were women (77%). The mean age was 51.4 years (SD 10.5). The majority (60%) of the patients in this sample were employed in caregiving and social work professions at the time of their infection. 

According to the body mass index (BMI), three-quarters of the patients were classified as overweight or obese. Vaccination status was documented in more than half of the cases (643; 56%). Of these, 87% had received the COVID vaccine, while 13% had not. At least two doses of a COVID vaccine had been administered to the majority of vaccinated patients (83%). Vaccination date information was available for 329 patients, of whom 117 (36%) had received at least one dose of the vaccine prior to infection. Most of the patients in this sample population were infected during the first wave of the pandemic (63%), when the wild-type SARS-COV-2 variant was dominant. A further 12%, 6.5% and 18.9% were infected during the Alpha, Delta and Omicron waves, respectively. SARS-CoV-2 reinfection was documented in 167 individuals (15% of the sample population), with 27 individuals (2%) reporting at least three confirmed infections. The majority of patients (98%) were unable to work during the course of the disease. Of these, 593 (52%) were on continuous sick leave from the time of their first infection until the PCC, while the remaining 430 individuals (37%) had intermittent periods of employment. One in two patients was still unable to work at the time of the examination. On average, the patients were on sick leave for 339 days (SD 221) due to persistent complaints. During the acute phase of COVID-19 infection (up to four weeks), the majority of patients experienced flu-like symptoms, including fever and chills (67%), coughing (66%), body aches (62%), headache (61%), and shortness of breath or difficulty breathing (61%). Symptoms with severe organ involvement (e.g., pneumonia, pulmonary embolism, renal failure or myocarditis) were documented in 97 (9%) patients. 60% of patients reported experiencing 5 to 9 different symptoms, while 14% reported 10 or more symptoms during the acute phase. Around 229 (20%) of the patients were hospitalized during the acute phase of COVID-19, with an average treatment duration of 14.6 days (SD 18.4). Among those hospitalized, 56 patients (25%) required intensive care, with an average stay of 19.5 days (SD 18.9). In this population, men (32%) were more likely to be hospitalized than women (17%). Among the 56 individuals who received intensive care treatment, 35 (64%) were still unable to work at the time of the study.

The majority (83%) had at least one pre-existing condition. The most commonly reported conditions were heart disease (48%), allergies (45%), lung disease (33%), mental illness (28%), and thyroid disease (27%). The majority of patients were multimorbid, with 55% of those with pre-existing conditions having three or more illnesses.

Documentation of long-term symptoms reveals that the most common issues are reduced physical capacity (95%), followed by concentration difficulties (79%), shortness of breath (69%), memory impairment (66%), and fatigue (66%). The vast majority (83%) of patients reported experiencing five or more persistent symptoms at the time of examination. Additionally, 81% of patients in the registry reported having already undergone inpatient and/or outpatient rehabilitation or received specialist consultations.

## Strengths and limitations

The registry included multidisciplinary data to study the long-term effects of COVID-19 and to optimize the diagnosis, treatment and rehabilitation of patients with PCS. The consolidation of several centers and interdisciplinary cooperation has resulted in a comprehensive and diverse database that enables a representative display of clinical practice and at the same time contributes to a more comprehensive picture of PCS. The extensive investigations carried out in the various medical disciplines can help to uncover previously unknown relationships and patterns. Furthermore, the registry data can be linked to the biobank data, which may provide further insights into biomarkers associated with PCS. In addition, the data obtained from longitudinal analysis will help to evaluate the effectiveness of the recommended rehabilitation measures. 

Despite the practical relevance, however, there are some limitations regarding the completeness and quality of the data. This is partly due to the different examination programs of the participating study centers and partly because the diagnostics and treatment are tailored to the individual symptoms of the patients. Moreover, different internal hospital documentation standards can lead to inconsistencies and potential inaccuracies in the data collected. As a result, the data are variable, and some analyses can only be carried out using subsamples. Data is therefore continuously checked for completeness, plausibility and consistency using standardized and automated procedures. Furthermore, no statements can be made about the total number of work-related COVID-19 cases, as the invitations for the PCC or an outpatient examination are issued depending on the assessment of the responsible accident insurance provider. It is also important to point out that different degrees of willingness exist among the patients for their data to be processed further.

## Conclusions

The post-COVID outpatient examination and the inpatient PCC are important measures for managing the recovery process of patients with work-related PCS. The multicenter clinical data obtained through these means can provide valuable information on the need for medical care and rehabilitation. By conducting in-depth analyses, we will be able to correlate subjective PCS symptoms with objective clinical data and thus contribute to a better understanding of PCS.

## Notes

### Competing interests

The authors declare that they have no competing interests.

### Ethical approval

This study was conducted after approval by the ethics committee of the Medical Faculty of Ruhr University Bochum (internal registration number 21-7317). 

### Funding

This work was financed and supported by the German social accident insurance provider for non-state institutions within the health and welfare service sectors (BGW). 

### Acknowledgments

We would like to thank the study staff and coordinators in the BG hospitals for their invaluable help with data collection, recording and advice: Heithem Ben Abdallah, Anke Dries, Carolin Fischer, Esther Herrmann, Siw Johannesen, Silke Keiser, Ina Kim, Yvonne Minkus, Jaqueline Noer, Yasothani Puwanesarasa, Melanie Röder, Kim Schleßelmann, Antje Spranger, Laura Suckert, Jana Thieben, Rebecca Trost, Leonie Zetzsch.

### Dedication

This publication is dedicated to Prof. Dr. Stephan Brandenburg, who organized the initial funding of the study. 

### Shared first autorship

Schwenkreis P and Kozak A contributed equally. 

### Authors’ ORCID

- Schwenkreis P:
https://orcid.org/0000-0003-2934-3923
- Kozak A:
https://orcid.org/0000-0003-4080-4524
- Gonschorek A:
https://orcid.org/0009-0007-2881-4945
- Rademacher C:
https://orcid.org/0009-0007-4947-3350
- Wefers J:
https://orcid.org/0000-0001-6597-6491
- Nienhaus A:
https://orcid.org/0000-0003-1881-7302
- Tegenthoff M:
https://orcid.org/0000-0001-9610-8451

## Figures and Tables

**Table 1:**
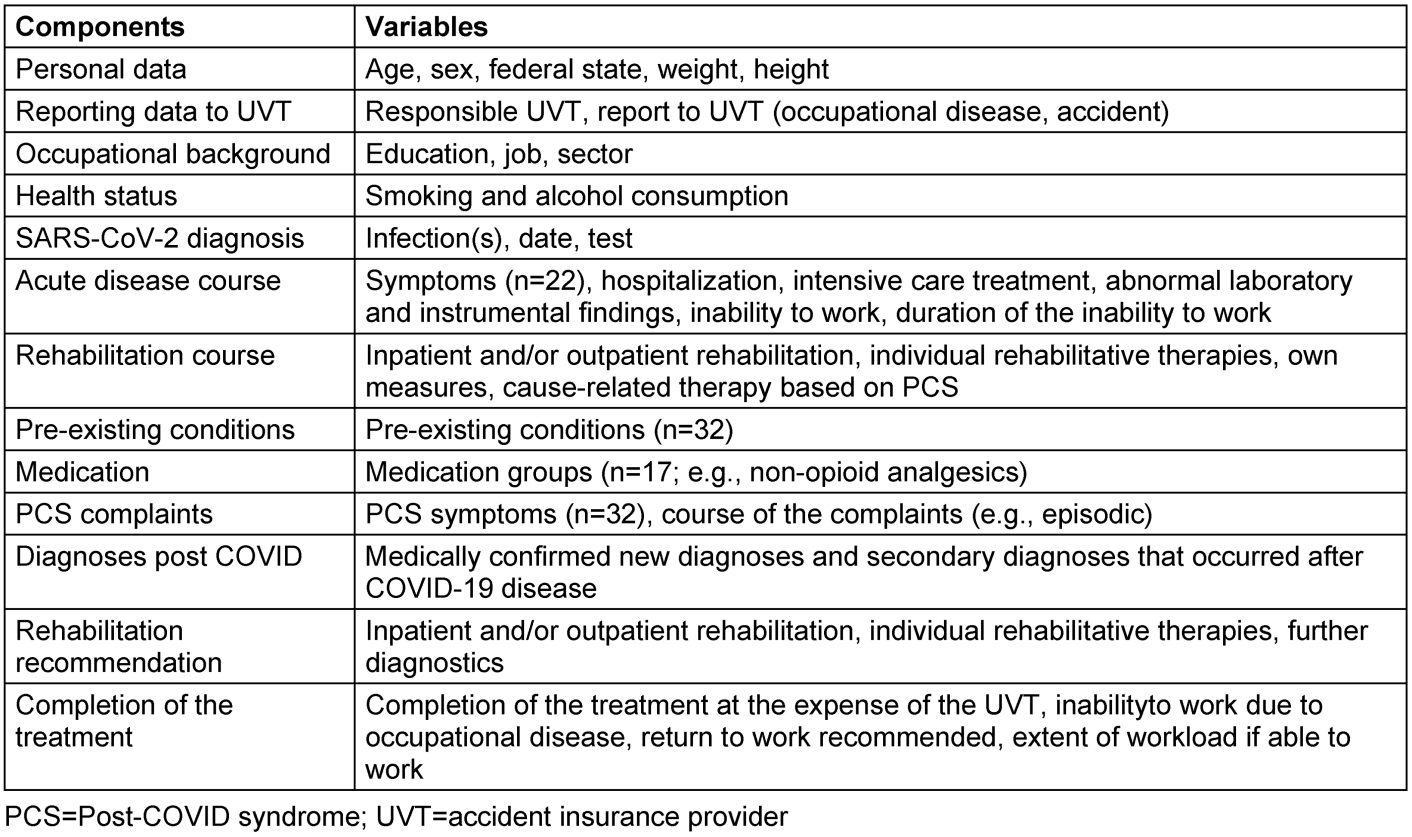
Overview of all components recorded as part of the PCC

**Table 2:**
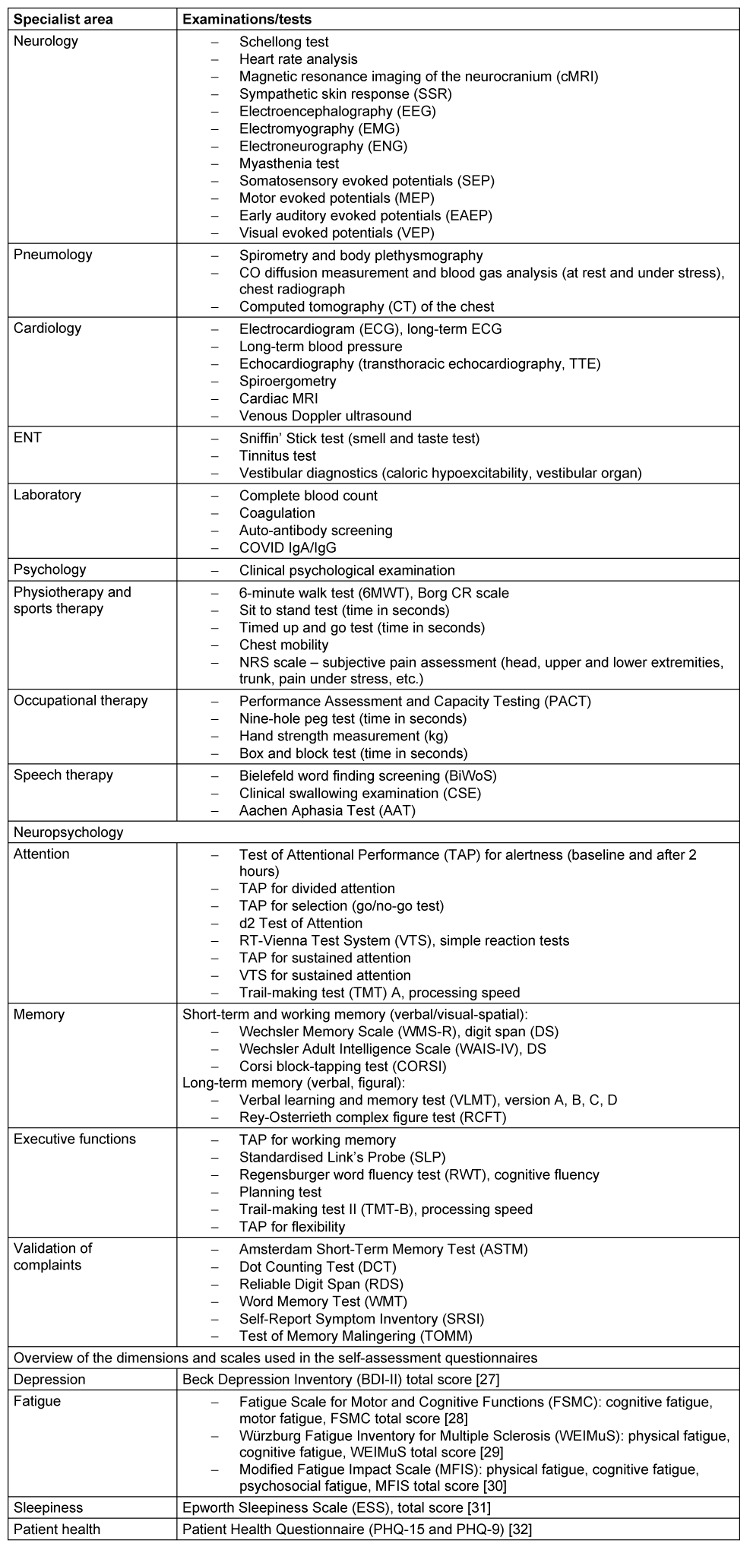
Overview of all clinical examination findings as part of the PCC

**Table 3:**
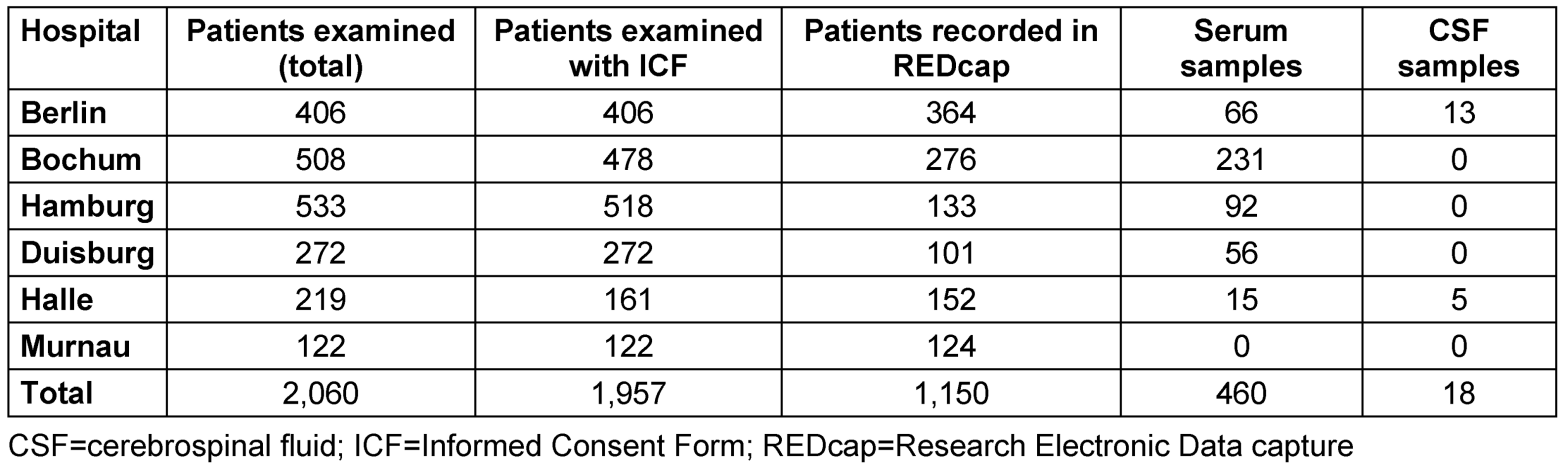
Inclusion of patients in the registry and biological materials repository

**Table 4:**
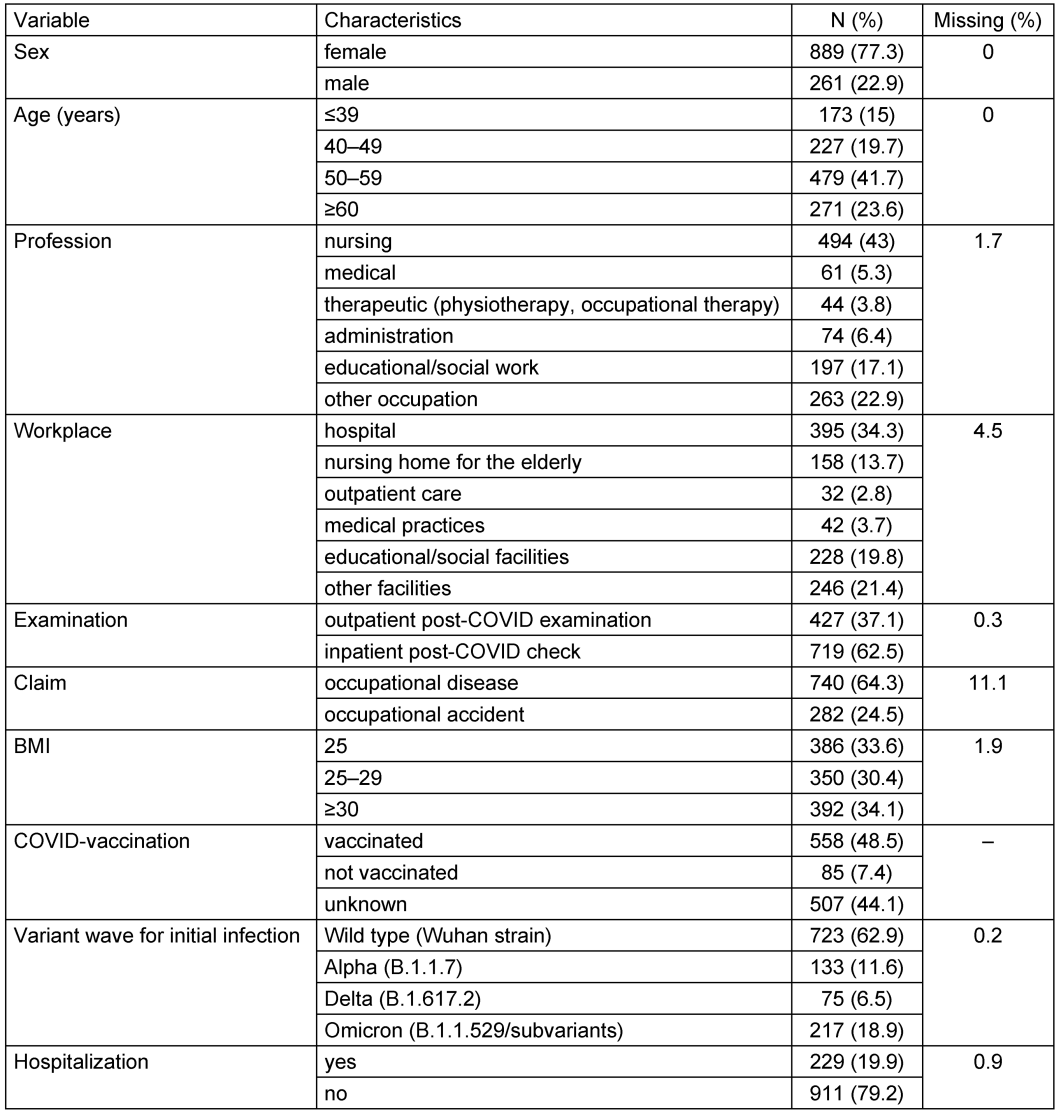
Description of the study population (n=1,150)
